# PAT1 inversely regulates the surface Amyloid Precursor Protein level in mouse primary neurons

**DOI:** 10.1186/s12868-015-0152-8

**Published:** 2015-03-07

**Authors:** Aysegul Dilsizoglu Senol, Lidia Tagliafierro, Léa Huguet, Lucie Gorisse-Hussonnois, Stéphanie Chasseigneaux, Bernadette Allinquant

**Affiliations:** INSERM UMR 894, Université Paris Descartes, Sorbonne Paris Cité, Faculté de Médecine, Paris, France; Department of Biochemistry, Biophysics and General Pathology, School of Medicine, Second University of Naples, Naples, Italy; Inserm, U1144, Paris, F-75006 France; Université Paris Descartes, UMR-S 1144, Paris, F-75006 France; Université Paris Diderot, UMR-S 1144, Paris, F-75013 France

**Keywords:** Amyloid precursor protein, PAT1 protein, Cell membrane, Protein trafficking, Neurons

## Abstract

**Background:**

The amyloid precursor protein (APP) is a key molecule in Alzheimer disease. Its localization at the cell surface can trigger downstream signaling and APP cleavages. APP trafficking to the cell surface in neurons is not clearly understood and may be related to the interactions with its partners. In this respect, by having homologies with kinesin light chain domains and because of its capacity to bind APP, PAT1 represents a good candidate.

**Results:**

We observed that PAT1 binds poorly APP at the cell surface of primary cortical neurons contrary to cytoplasmic APP. Using down and up-regulation of PAT1, we observed respectively an increase and decrease of APP at the cell surface. The increase of APP at the cell surface induced by low levels of PAT1 did not trigger cell death signaling.

**Conclusions:**

These data suggest that PAT1 slows down APP trafficking to the cell surface in primary cortical neurons. Our results contribute to the elucidation of mechanisms involved in APP trafficking in Alzheimer disease.

## Background

Amyloid Precursor Protein (APP) is a key molecule in Alzheimer disease (AD) by the generation of its metabolites [1,2]. APP at the cell surface is required for its cleavages. Beside its cleavages, APP can receive signals transduced through Go to induce neuronal migration and outgrowth, synapse differentiation at the neuro-muscular junction in Drosophila or cell death [3-10]. The generation of APP cleavages following this signaling has not been reported. Some molecules like reelin, able to induce neurite outgrowth in primary neurons, promote a rapid traffic of APP to the cell surface [11]. Interestingly, this increase of APP at the cell surface is required for reelin-induced neurite outgrowth suggesting that APP at the cell surface triggers some signaling in this model [11]. Consequently, APP trafficking to the cell surface appears crucial, but its mechanisms remain poorly investigated. APP is a fast axonal transported protein along the microtubules, involving kinesin and Rab3 GTPase activity [12-21]. The role of APP binding with its partners either in the N or in the C-terminal domains has been poorly investigated and some of these interactions could be involved in APP trafficking. The neuronal sorting protein-related receptor SorLA/LR11, a retromer-associated protein, interacts with the APP carbohydrate-linked domain and retains APP in Golgi [22,23]. Sortilin, a VPS10p-domain receptor, related to SorLA interacts with both the extracellular and intracellular domains of APP in neurites and regulates APP trafficking to lysosomes and lipid rafts [24]. Mint proteins bind APP and ADP-ribosylation factors suggesting that Mint proteins can regulate vesicular trafficking of APP and its endocytosis [25]. Recently, Mint3 adaptor has been shown necessary for export of APP from the Golgi to a LAMP1+ compartment [26]. AP-4 has been shown to be important for the APP export from the Golgi in HeLa cells [27]. Most of APP partners are localized in the distal part of the C-terminal domain, probably due to its structure having two type I reverse turns, the first one at the TPPEE sequence [28,29]. Consequently, the APP cytoplasmic juxtamembrane domain may be less accessible to partners unless it is unmasked after caspase cleavage. The PAT1 (Protein interacting with APP tail 1) also called APPBP2 (Amyloid protein-binding protein 2) or Ara67 interacting with the APP cytoplasmic juxtamembrane domain and bearing repeat homologies to kinesin light chains has been reported to increase APP trafficking to the cell surface when co-transfected with APP in COS cells [30]. PAT1 is expressed in many human tissues at variable levels and in many cell lines [31]. More recently, a polymorphism of the PAT1/APPBP2 gene was shown at the origin of sequence differences in PAT1, Ara67 and APPBP2 [32]. PAT1a is 99% identical to PAT1 and completely identical to APPBP2. Ara67 is 99.6% identical to PAT1 [31,32]. PAT1a when overexpressed in neuroblastoma cells with APP, induces an increase of APP at the cell surface [32]. We have previously shown that overexpression of the APP-PAT1 binding domain in the cytoplasm of primary neurons through the help of an internalization vector, leads to a very rapid increase of APP at the cell surface bound to a low amount of PAT1 triggering a cell death signal [33]. In this model, PAT1 was trapped by the APP-PAT1 binding domain disrupting PAT1 from its interactions with endogenous APP, suggesting that the binding of PAT1 with endogenous APP prevents its trafficking to the cell surface. In order to address the role of PAT1 in APP trafficking at the cell surface in primary neurons, we down and up-regulated PAT1 and observed that APP at the cell surface is dependent on a low amount of PAT1 suggesting that the binding of PAT1 to APP keeps APP in the cytoplasm.

## Results

### Colocalization of APP and PAT1 is poorly present at the cell surface of neurons

**Figure 1:**
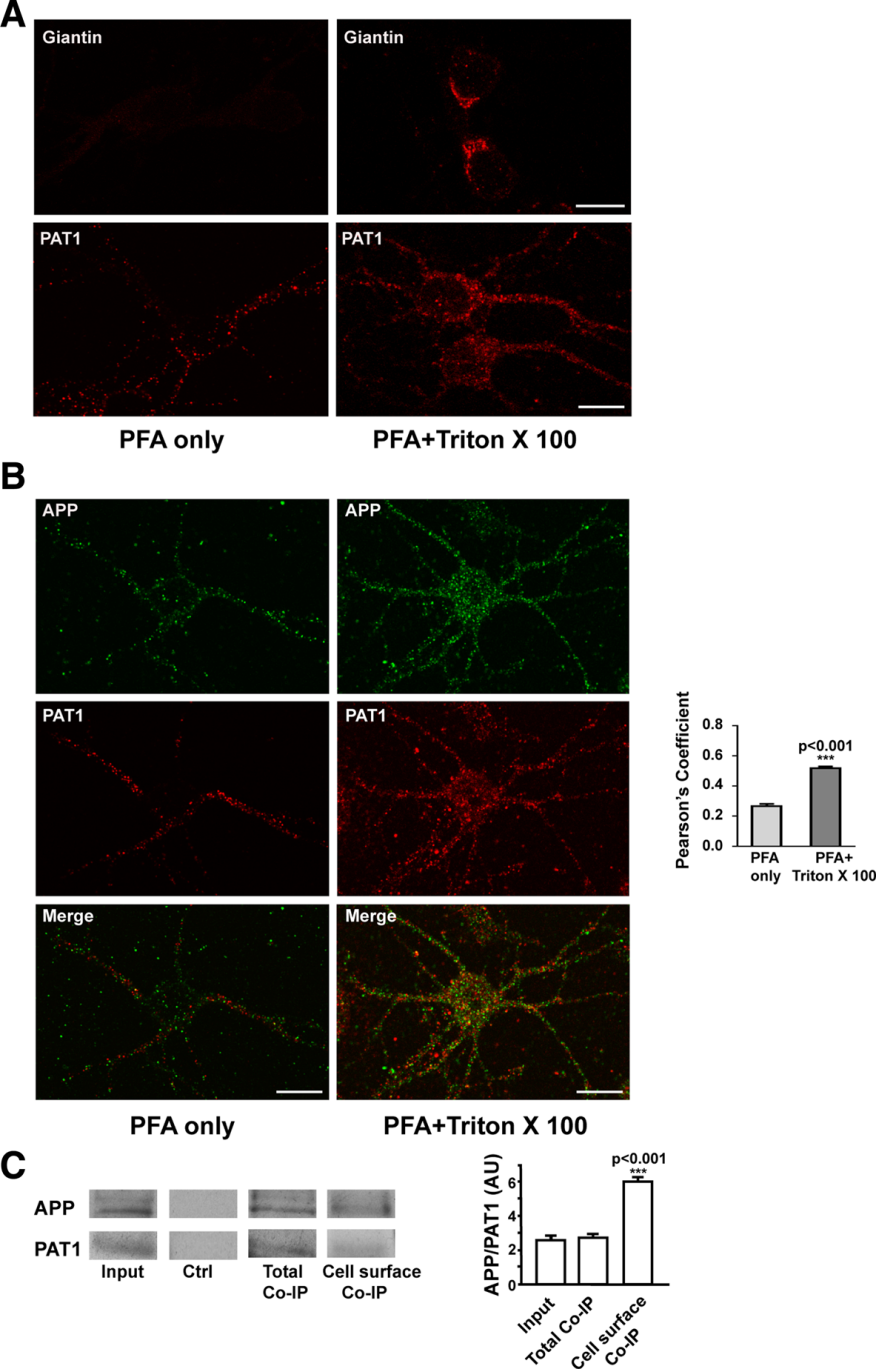
**APP and PAT1 colocalize poorly at the cell surface of primary neurons. A)** Neurons at 5 DIV were fixed in PFA 4% for 30 min (left panel). After fixation, an additional permeabilization in 0.2% Triton X 100 was performed (right panel). In both cases cells were immunolabeled for Giantin or PAT1. Immunocytochemistry was analyzed by confocal microscopy. One representative immunocytochemical staining out of 4 independent experiments is presented. Scale bar: 10 μm. **B)** APP and PAT1 double immunolabeling was performed in neurons at 5 DIV fixed with PFA 4% only (left panel) and followed by 0.2% Triton X 100 (right panel). Anti-APP Cter polyclonal and monoclonal PAT1 antibodies were used as primary antibodies. Pearson’s coefficient was evaluated following confocal microscopy analyses and quantifications in Volocity software. Data presented are the mean ± SEM of 4 independent experiments. **C)** Ratio of APP to PAT1 before (Input) and after immunoprecipitation of APP in total extracts (Total Co-IP) and after cell surface biotinylation (Cell surface Co-IP). Immunoprecipitation of APP was performed with the anti-APP Cter polyclonal antibody. 10.10^6^ cells at 6 DIV were used for each condition. 40 μg of cell extracts before immunoprecipitation were loaded (Input). Control immunoprecipitation in absence of primary antibody is presented (Ctrl). Detection of APP in western blotting was performed using the anti-APP-Nter A4 antibody. Data presented are the mean ± SEM of 3 independent experiments. Representative immunoblot and histogram of the ratio of APP to PAT1 in arbitrary units (AU) are presented.

We first observed in primary cortical neurons that paraformaldehyde (PFA) fixation at 4% caused little permeabilization at the cell surface allowing some PAT1 detection under the cell surface while additional permeabilization by 0.2% Triton X 100 allowed to detect more PAT1 in the cytoplasm (Figure 1A). The Giantin a Golgi marker is detected after additional permeabilization with 0.2% Triton X 100 only.

PAT1 was detected close to the cell surface of soma and neurites. Double immunostaining for APP and PAT1, performed after 4% PFA fixation only, reveal little co-localization close to the cell surface contrary to what observed in the cytoplasm when the fixed cells were then permeabilized with 0.2% Triton X 100. Pearson’s coefficient shows a significant difference (p < 0.001) between the colocalization of APP and PAT1 detected after fixation in PFA only and the colocalization observed when the fixation is followed by an additional permeabilization in Triton X 100 (Figure 1B).

Immunoprecipitation of APP from total neuronal extracts show co-immunoprecipitation of PAT1 as expected. In that case the ratio of APP to PAT1 (Mean ± SEM: 2.7 ± 0.2) was close to that observed in lysates before immunoprecipitation (Mean ± SEM: 2.6 ± 0.1). After cell surface biotinylation this ratio was significantly higher (Mean ± SEM: 6.0 ± 0.2) (Total Co-IP *vs* Cell surface Co-IP: p < 0.001) (Figure 1C). These data confirm that at cell surface the amount of PAT1 attached to APP is lower than in the cytoplasm.

### Down and up-regulation of PAT1 respectively increases and decreases APP at the cell surface of neurons

**Figure 2:**
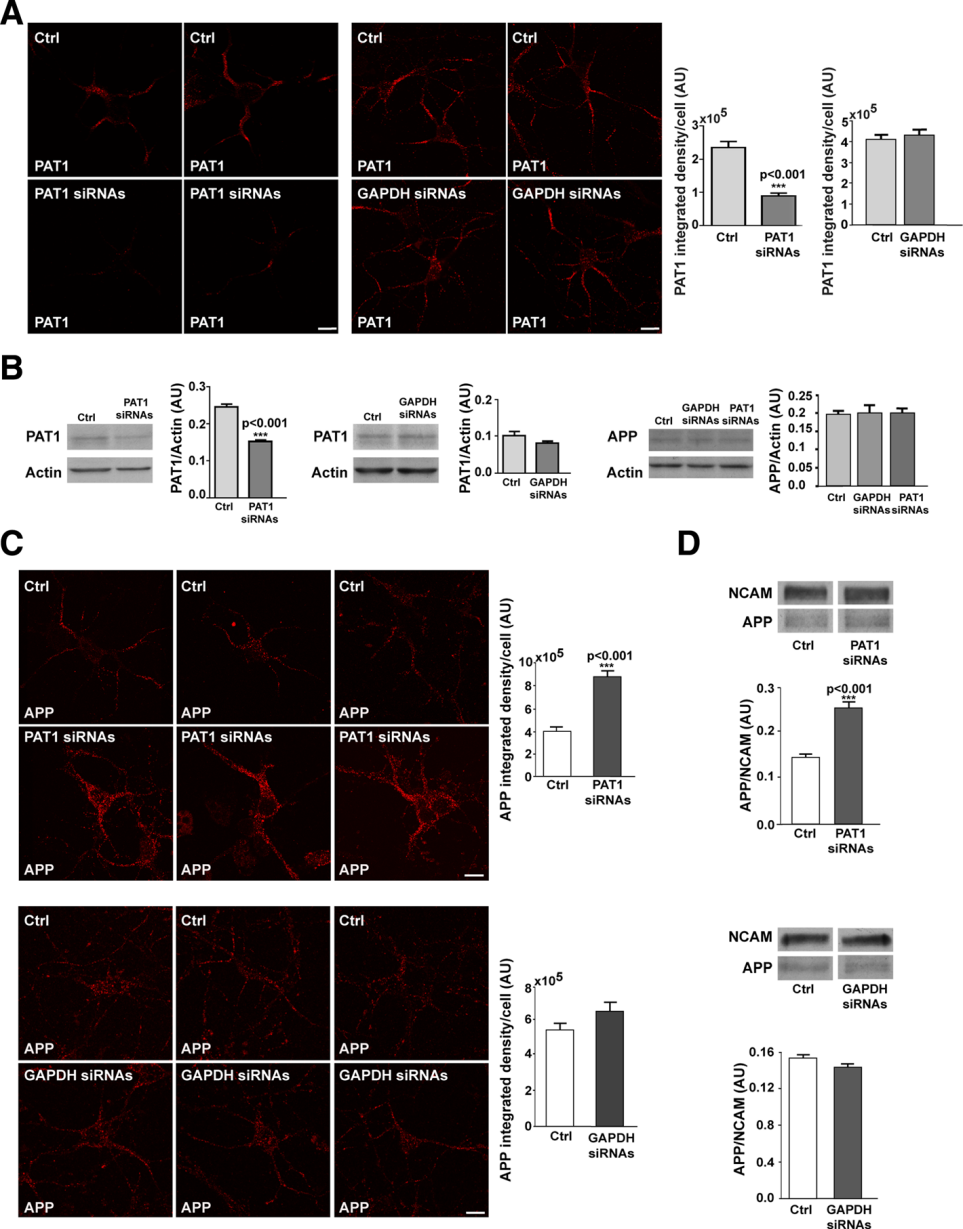
**Down-regulation of PAT1 increases APP at the cell surface of primary neurons.** Neurons at 2 DIV were treated with either PAT1 siRNAs or GAPDH siRNAs comparatively to control cells (Ctrl) in absence of treatment. **A-B:** After 66 h the cells were fixed and processed for PAT1 immunocytodetection **(A)** or extracted for western blots **(B). (A)** Immunocytochemistry was analyzed by confocal microscopy and quantified by Image J. Data are expressed in integrated density/cell in arbitrary units (AU). Two representative immunolabelings of PAT1 of each condition are presented. **(B)** 40 μg of cell extracts were loaded for western blotting. The level of PAT1 or of APP reported to actin was expressed in arbitrary units (AU). Data in **A** and **B** are the mean ± SEM of 4 independent experiments and of 3 experiments for right panel in B. **C-D:** After 66 h of PAT1 siRNAs or GAPDH siRNAs, the cells were fixed by and processed for APP immunodetection **(C)** or for cell surface biotinylation **(D). (C)** APP immunocytochemistry was analyzed by confocal microscopy and quantified with Image J. Data are expressed in integrated density/cell in arbitray units (AU). Three representative images of each condition are presented. **(D)** Cell surface biotinylation was performed on 10^6^ cells. NCAM was used as internal control of membrane loading for cell surface biotinylation. The level of APP in biotinylated membranes was reported to NCAM and expressed in arbitrary units (AU). Data are the mean ± SEM of 3 independent experiments. In the whole figure immunocytochemistry was performed using the anti-APP-Nter A4 antibody. The anti-APP Cter polyclonal antibody was used for western blotting in 2B (right panel) and the anti-APP-Nter A4 antibody was used in 2D. Scale bar: 10 μm.

In order to investigate if PAT1 levels can modulate the trafficking of APP to the cell surface we down and up-regulated PAT1. Down-regulation of PAT1 was performed by the help of specific siRNAs. The decrease of PAT1 level in primary neurons was checked 66 h after PAT1 siRNAs addition both by immunocytochemical staining for PAT1 without Triton X 100 and immunoblots from cell extracts (Figure 2A-B). A significant decrease of PAT1 level was observed in primary neurons treated with PAT1 siRNAs for 66 h comparatively to control (ctrl) cells in absence of treatment (ctrl *vs* PAT1 siRNAs: p < 0.001) while no difference of PAT1 levels was observed using GAPDH siRNAs, as control siRNAs (ctrl *vs* GAPDH siRNAs: NS) (Figure 2A-B). The down-regulation of PAT1 or GAPDH did not modify the level of total APP in neuronal extracts (Figure 2B).

**Figure 3:**
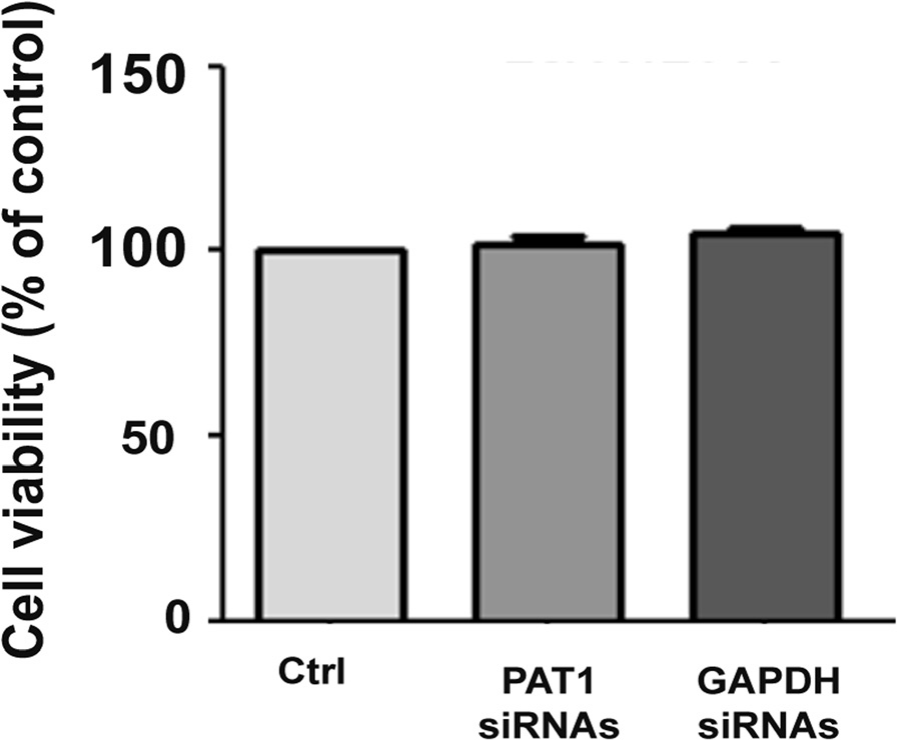
**Cell viability of neurons after treatment by PAT1 siRNAs or GAPDH siRNAs.** Cells in absence of treatment (Ctrl), and after 66 h of PAT1 siRNAs or GAPDH siRNAs were tested for cell viability using the Cell Titer-Glo-Luminescent Cell Viability kit. Data are expressed in % of control. Data are the mean ± SEM of 2 independent experiments.

The localization of APP in cortical neurons was checked in primary neuronal culture treated with PAT1 siRNAs for 66 h. Confocal analyses of immunocytochemical APP detection in absence of Triton X 100 showed a significant increase of APP at the cell surface in presence of low levels of PAT1 (ctrl *vs* PAT1 siRNAs: p < 0.001). This increase of APP was present both in soma and neurites (Figure 2C, upper panel). Cell surface biotinylation performed after 66 h of PAT1 siRNAs treatment confirmed this APP increase at the cell surface (ctrl *vs* PAT1 siRNAs: p < 0.001) (Figure 2D, upper panel). As total APP was not modified in PAT1 siRNAs conditions (Figure 2B), these data suggest that the APP increase at the cell surface is induced by low levels of PAT1 and not by changes in total APP levels. No difference of APP at the cell surface was observed when the neurons were treated with GAPDH siRNAs (ctrl *vs* GAPDH siRNAs: NS) (Figure 2C-D, lower panels). We did not observe cell death in neurons treated with PAT1 siRNAs, nor with GAPDH siRNAs, suggesting that the increase of APP at the cell surface, in conditions of PAT1 low levels did not induce cell death (Figure 3).

**Figure 4:**
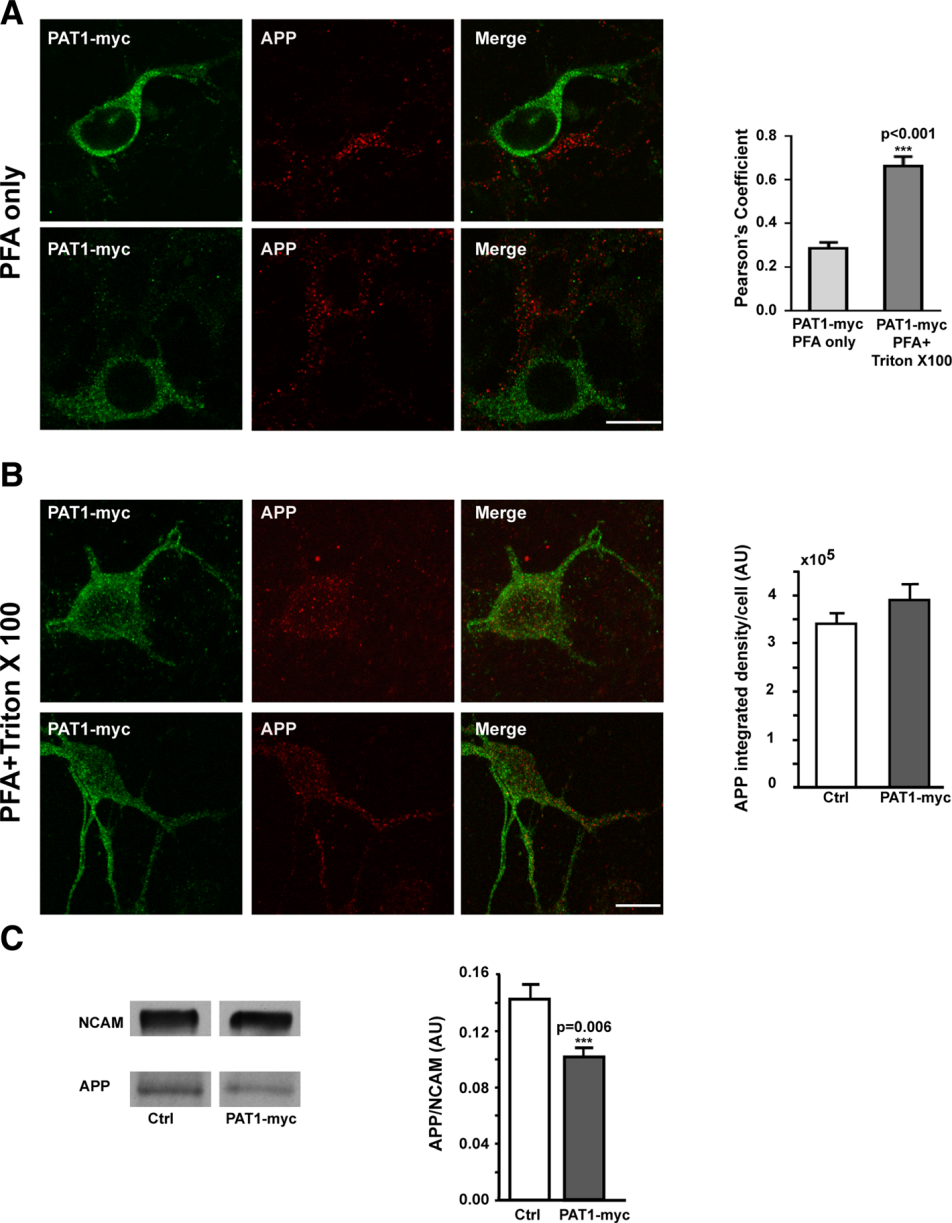
**Up-regulation of PAT1 in primary neurons results in a strong colocalization with APP in the cytoplasm but not at the cell surface.** A-B. PAT1-myc was overexpressed by transfection in primary neurons at 5 DIV. 24 h later cells were fixed by PFA 4% only **(A)** or followed by 0.2% Triton X 100 **(B)**. Immunolabeling for PAT1-myc (Alexa-488) and endogenous APP (Cy3) was performed and analyzed by confocal microscopy. Two representative images in each condition are presented. In **(A)** immunolabeling was performed using the anti-myc tag polyclonal #06-549 and the anti-APP-Nter A4 antibody. In **(B)** the anti-myc tag MABE282 and the anti-APP-Cter polyclonal antibodies were used for immunolabeling. Quantification of colocalization using Volocity software was expressed by Pearson’s coefficient (upper panel). Data presented are the mean ± SEM of 3 independent experiments. Quantification of total APP in conditions of 0.2% Triton X 100 was performed in 23 transfected and 42 non-transfected cells (Ctrl) out of 3 independent experiments. Data are expressed in integrated density / cell in arbitrary units (AU) and represents the mean ± SEM (lower panel). Scale bar: 10 μm. **C)** Cell surface biotinylation was performed on PAT1-myc overexpressed cells comparatively to control cells (Ctrl). Experiments were performed on 10.10^6^ cells 24 h after transfection. NCAM was used as internal control of membrane loading for cell surface biotinylation. The level of APP in biotinylated membranes was reported to NCAM and expressed in arbitrary units (AU). Data are the mean ± SEM of 3 independent experiments. Western blotting were performed using the anti-APP-Nter A4 and the anti-NCAM antibodies.

Conversely, up-regulation of PAT1 was performed by transfection of PAT1-myc in primary cortical neurons. Then, the cells were fixed 24 h later, and processed for myc and APP double immunolabeling. Cells overexpressing PAT1-myc have little APP at the cell surface as observed by immunolabeling performed after PFA fixation in absence of any further permeabilization (Figure 4A). However when immunolabeling was performed in conditions of additional permeabilization with Triton X 100, more endogenous APP within the cytoplasm colocalized with PAT1-myc in transfected cells (Figure 4B), as indicated by Pearson’s coefficient. A significant increase of Pearson’s coefficient (p < 0.001) was observed in conditions of additional permeabilization comparatively to conditions of PFA fixation only (Figure 4A-B). Cell surface biotinylation of transfected neurons showed a significant lower ratio of APP/NCAM than in the control conditions, confirming immunocytochemical data (Ctrl vs PAT1-myc: p = 0.006) (Figure 4C). The total APP level detected in transfected cells by immunolabeling in conditions of additional permeabilization with Triton X 100, was not significantly different to that of non-transfected cells suggesting that the overexpression of PAT1-myc does not reduce the total level of APP (Figure 4B). Consequently, the decrease of APP at the cell surface is induced by the overexpression of PAT1-myc and not by changes in APP total levels under these conditions.

The whole data suggest that PAT1 slows down APP trafficking to the cell surface.

## Discussion

Using down and up-regulation of PAT1 in primary neurons, we show here that PAT1 is involved in APP trafficking to the cell surface.

PAT1 down-regulation through siRNAs causes an increase of APP at the cell surface while up-regulation of PAT1 shows very little if any APP close to the plasma membrane. PAT1 down-regulation experiments fit with what we observed when PAT1 was mislocalized by overexpressing the APP-PAT1 binding domain [33]. In that case, PAT1 was attached to the APP-PAT1 binding domain in the cytoplasm detrimental to endogenous APP. The binding of endogenous PAT1 to the APP-PAT1 domain internalized through a cell permeable peptide led to a rapid increase of APP at the cell surface, as early as one hour. In this case APP migrating at cell surface had a very low level of PAT1, while the internalized APP-PAT1 binding domain was also able to interact in a tripartite interaction with APP and PAT1 in the cytoplasm. Indeed, we showed that this increase of APP and of its homolog Amyloid Precursor like Protein 2 (APLP2) at the cell surface induced a cell death signal with downstream events like translocation of SET from the nucleus to the cytoplasm. In this model the level of PAT1 still attached to APP at the cell surface was lower than in control conditions, and probably lower than in PAT1 siRNAs conditions as well. In addition, we cannot exclude that other partners able to interact with PAT1 were probably mislocalized with PAT1 and could act in the cell death signal induced by the increase of APP at the cell surface. In the present study, in conditions where PAT1 was down-regulated by siRNAs, APP reached rapidly the cell surface, as if it was liberated from some anchor in the cytoplasm but did not induced cell death, probably because about 50% of PAT1 was still present and not mislocalized. Down-regulation using siRNAs remains more stable contrary to the very transient efficiency of antisense oligonucleotides strategy which is useful for a rapid signal induced at the time of its highest efficiency, as we observed with the APP-PAT1 binding domain peptide but which can mask some events [33]. We don’t know precisely at what level of PAT1 within the neuron, APP starts to migrate to the cell surface. In control conditions, we observed here that the amount of PAT1 interacting with APP at the cell surface is lower than with the total level of APP interacting mainly within the cytoplasm, meaning that at the cell surface PAT1 is poorly required.

The down-regulation data obtained with PAT1 siRNAs fit with the PAT1 up-regulation results, where little APP reached the cell surface while it is present deeply in the cytoplasm co-localizing with PAT1 probably by direct interaction. Our data fit with what observed with Ara67/PAT1 which binds to androgen receptor in the cytoplasm of cell lines overexpressing these two partners suppressing the migration of androgen receptor to the nucleus and its transactivation [31].

Whether the ligands acting on a specific receptor and inducing an increase of APP at the cell surface are related to a disruption of the interaction with PAT1 remain to be elucidated. In the reelin signaling pathway the increase of APP at the cell surface was also observed in presence of high levels of Disabled-1, the reelin signaling molecule, able to bind the APP cytoplasmic distal domain [34]. As PAT1 binds the APP cytoplasmic juxtamembrane domain, in pathological conditions where there is an increase of the neuronal APP cleaved in its cytoplasmic domain by a caspase [35-38], one can wonder if the uncleaved APP migrates rapidly to the cell surface to trigger a cell death signal stimulating the deleterious effect.

Previous report has shown that the anterograde transport of APP was independent of its C-terminal domain but required the Rab3GTPase activity for recruitment of kinesin 1C to vesicles containing APP allowing to interact with microtubules [21]. Nothing is known if Rab3GTPase activity is necessary for the retrograde APP trafficking as well as its trafficking to the cell surface. One cannot exclude that PAT1 having several repeats of kinesin light chains and interacting with APP prevents the APP containing vesicles to move by competing with kinesin 1C in the interaction with microtubules [21]. Our results contradict the data obtained in APP and PAT1 or PAT1a double transfected COS and neuroblastoma cells [30,32]. PAT1 binding domain to APP has not been determined and as PAT1/PAT1a contains several repeats, it is possible that PAT1/PAT1a acts in a multipartite APP interaction. In case of double transfected cells it would have been interesting to evaluate the ratio of PAT1/PAT1a to APP at the cell surface and within the cytoplasm. The modulation of the level of endogenous PAT1 in primary differentiated neurons could represent more physiological conditions.

In addition to cell signaling, increases of APP at the cell surface may induce APP cleavages. However, we could not establish an increase of APP cleavages either in alpha or beta secretase in PAT1 down-regulation conditions (not shown). This may be explained by low detectable amyloid products in our primary neurons.

Since PAT1 poorly binds APP at the cell surface, it is possible that PAT1 may have other partners and function. The role of PAT1 in pathology has not been elucidated. It is highly expressed in breast cancers with poor diagnosis [39], in ovarian adenocarcinoma tumors [40], but also in neuroblastomas [41], and desmoplastic medulloblastoma cerebellar tumors [42]. PAT1 binds also to the C-terminal domain of the herpes simplex virus type 1 Us11 gene product and probably this interaction plays a role in the intracellular movement of viral components [43]. Changes in PAT1 expression in Alzheimer disease have not been yet investigated but could have consequences in APP signaling and cleavages.

## Conclusion

In primary cortical neurons APP is poorly associated to its partner PAT1 at the cell surface contrary to what observed in the cytoplasm. We used down and up-regulation of PAT1 in primary neurons and observed that APP respectively increases and decreases at the cell surface. The modulation of APP at the cell surface through PAT1 levels can represent a way to control the cell surface signaling events and APP cleavages. These data contribute to the understanding of mechanisms involved in Alzheimer disease.

## Methods

### Ethics statement

The protocols of animal anesthesia were performed according to the recommendations of the French National Committee (87/848) and European Economic Community (86/609/EEC) and were approved by the local ethics committee (Direction départementale des services vétérinaires de Paris, service de la protection et santé animales et de la protection de l’environnement).

### Primary cortical neurons

Primary cortical neurons were prepared from E16 mouse embryos from Swiss strain mice as previously described [33]. Briefly, dissociated cells were plated on polyornithine-coated glass coverslips for immunochemistry and on plastic dishes for cell surface biotinylation, immunoprecipitation and cell death quantification. Cells were plated at 15 × 10^4^ cells/cm^2^ for control and siRNAs experiments and at 30 × 10^4^ cells/cm^2^ for transfection experiments. Cell death experiments were performed on ELISA coated dishes at 20 × 10^3^ cells/well.

### Down-regulation of PAT1

Small interfering RNA duplexes (siRNAs) were used for down-regulation of PAT1 siRNAs (J-059477-10 and J-059477-12, Thermo Fischer Scientific, St Leon-Rot, Germany) at the final concentration of 100 nM. GAPDH siRNAs were used as siRNA negative control (AM4624, Thermo Fisher Scientific). Briefly, siRNAs were internalized into primary neurons with the help of penetratin (MP Biomedicals, Illkirsch, France) in a ratio of 1 to 10 (siRNAs to penetratin) as previously described [33]. siRNAs were mixed to penetratin at room temperature for 30 min, before their addition in the medium. Cells were incubated for 4 h at 37°C. Then half fresh medium was added and left at 37°C for 66 h.

### Human PAT1 cloning

Human total brain RNA (Ozyme, Saint-Quentin-en-Yvelines, France) was converted into cDNA by high capacity cDNA reverse transcription kit (Life Technologies, Saint Aubin, France) following the manufacturer’s instructions. Human PAT1 full length coding sequence was generated by forward primer (Eurofins MGW Operon, Les Ullis, France): 5′-ATGGCGGCCGTGGAACTAGA-3′ and reverse primer: 5′-AATGTCGAGGGACCGAGCTGC-3′. The amplified fragment was used as a template to amplify the same region with restriction sites for EcoRI and NotI respectively, by the forward primer: 5′-TATCGAGAATTCATGGCGGCCGTGGA-3′ and reverse primer: 5′-ACCGAGCTGCGGCCGCTTAAT-3′ in order to be cloned into pcDNA3.1/myc-His vector (Life Technologies).

Plasmids were transformed into *E.Coli* strain XL10-Gold ultracompetent cells (Agilent Technologies, Les Ullis, France). Positive colonies were selected by PCR and the generated plasmid was sequenced.

### Antibodies

The supernatant of the hybridoma cell line mAb26 [30] was used as the antiserum against PAT1. It was used diluted to 1/20 for immunocytochemistry and to 1/200 for western blotting.

The other antibodies were purchased and diluted as follows:

Rabbit polyclonal anti-Giantin (Abcam Cat# ab24586 RRID:AB_448163, Cambridge, UK) diluted to 1/5000 for immunocytochemistry; the monoclonal anti-APP-Nter anti-Alzheimer Precursor Protein A4 (EMD Millipore Cat# MAB348 RRID:AB_94882, Saint-Quentin en Yvelines, France) diluted to 1/100 for immunocytochemistry, and to 1/1000 for western blotting; anti-myc tag (EMD Millipore Cat# MABE282 RRID:AB_112045219E10,) diluted to 1/200, anti-myc tag rabbit polyclonal (EMD Millipore Cat#06-549 RRID: AB_310165) diluted to 1/200, anti-APP-Cter polyclonal antibody generated in rabbit [44] diluted to 1/500 for immunocytochemistry; anti-Actin mouse monoclonal antibody (EMD Millipore MAB 1501R Cat# MAB1501 RRID:AB_2223041), diluted to 1/10000 for western blotting; anti-NCAM (Sigma-Aldrich Cat# C9672 RRID:AB_1079450, l’Isle d’Abeau Chesnes, France) diluted to 1/2000 for western blotting.

Secondary antibodies used for immunocytochemistry were Donkey anti-rabbit cy3 (Cat# 711-166-152, Jackson Immuno-research, Interchim, Montluçon, France) diluted to 1/500, Donkey anti-mouse cy3 (Cat# 715-166-151 Jackson Immuno-research, Interchim), diluted to 1/200 and Donkey anti-mouse Alexa 488 (Cat# 715-546-151 Jackson Immuno-research, Interchim) diluted to 1/200.

### Cell surface biotinylation and western blotting

Cell surface biotinylation after siRNAs addition were carried out on 10^6^ primary neurons at 5 days in vitro (DIV) as previously described [33]. For PAT1-myc overexpressed cells, cell surface biotinylation experiments were performed on 10 × 10^6^ cells at 6 DIV, 24 h after transfection. Briefly, the cells were washed with cold PBS-glucose and incubated for 30 min on ice with sulfo-NHS-biotine (Pierce) at 35 μg/ml. Then the cells were washed, scrapped in PBS sucrose 0.32 M, homogenized and centrifugated at 16,000 × g for 30 min at 4°C. The particulate pellet was solubilized and added to streptavidin sepharose beads overnight at 4°C. After 4 washings the beads were eluted in Laemmli buffer.

Western blotting was performed as previously described [33]. Briefly SDS-PAGE in 7.5 or 10% acrylamide precast gels (Invitrogen, Life Technologies) were performed. After electrophoresis, electrotransfer was carried out onto PVDF membranes. Immunodetection was processed as previously described. Secondary goat anti-mouse horseradish peroxidase antibody (Biorad, Cat# 170–6516, Marnes-la-Coquette, France) were used and proteins were detected by chemiluminescence. ECL (Fischer Scientific, Illkirsch, France) films were quantified by densitometry.

### Immunoprecipitation

Neurons at 6 DIV were rinsed two times in PBS and harvested in IP buffer: Hepes 10 mM, NaCl 150 mM, CaCl_2_ 2 mM, 1% Triton X 100, protease inhibitors (Complete EDTA free, Roche Diagnostics, Mannheim, Germany). After 15 min at room temperature under agitation, the lysate was centrifuged at 16,000 × g for 45 min at 4°C. The supernatant was kept and pre-cleared with protein A Sepharose for 1 h at 4°C. After centrifugation at 800 g for 10 min at 4°C, the anti-APP-Cter antibody was added to the supernatant and left overnight at 4°C under rotate agitation. Then protein A sepharose was added and left 2 h at 4°C under rotate agitation. The beads were then washed two times in IP buffer and a third time in IP buffer without Triton X 100. The beads were then eluted in Laemmli buffer and the proteins loaded in 7.5% acrylamide gels for SDS-PAGE followed by immunoblots for APP and PAT1. A negative control of the immunoprecipitation was performed in parallel in the absence of primary antibody.

### Up-regulation of PAT1

PAT1-myc plasmid was transfected into 5 DIV cortical neurons (3 x 10^5^ cells per coverslip) via Lipofectamine™ 2000 Reagent (Life Technologies) following the manufacturer’s instructions.

### Immunocytochemistry

Immunocytochemistry was performed on 4% paraformaldehyde (PFA) fixed cells for 30 min at room temperature. After 3 washes in PBS, in about half of the experiments the cells were permeabilized with 0.2% Triton X 100 in saturation buffer (10% fetal calf serum in PBS) for 1 h at 37°C. They were then incubated with the primary antibody for 1 h at 37°C and after 3 rinses in PBS, cells were incubated with anti-rabbit cy3 or anti-mouse cy3 for 1 h at 37°C. After 3 rinses in PBS the coverslips were mounted in a medium containing DAPI.

Immunofluorescence was examined with a TCS SP5 confocal imaging system equipped with DPSS 561 nm and HeNe 633 nm lasers (Leica Microsystems, Mannheim, Germany). Eight bit digital images were collected in sequential mode with a x63 plan Apochromat oil immersion objective, a numerical aperture of 1.4, a zoom of 3 and the pinhole size “airy 1”. Laser intensities and imaging settings were adjusted for each experiment and subsequently kept constant. Microscopy was performed at the PICPEN platform (INSERM, UMR 894).

Pearson’s coefficient was evaluated using Volocity software on confocal images.

Integrated density measurements were performed by Image J software.

### Cell death quantification

Cell death quantification was carried out 66 h after siRNA addition using the Cell Titer-Glo-Luminescent Cell Viability kit (Promega, Charbonnières, France) according to the manufacturer’s instructions.

### Statistical analysis

Statistical analyses were performed respectively using the analysis of variance (ANOVA) and post-hoc Scheffe’s tests (Statview).
